# *apex*: phylogenetics with multiple genes

**DOI:** 10.1111/1755-0998.12567

**Published:** 2016-08-12

**Authors:** Thibaut Jombart, Frederick Archer, Klaus Schliep, Zhian Kamvar, Rebecca Harris, Emmanuel Paradis, Jérome Goudet, Hilmar Lapp

**Affiliations:** ^1^ Department of Infectious Disease EpidemiologyMRC Centre for Outbreak Analysis and ModellingSchool of Public HealthImperial College LondonSt Mary's Campus, Norfolk PlaceLondonW2 1PGUK; ^2^ Southwest Fisheries Science CenterNMFS‐NOAA8901 La Jolla Shores DriveLa JollaCA92037‐1508USA; ^3^ Department of BiologyUniversity of Massachusetts Boston100 Morrissey BlvdBostonMA02125‐3393USA; ^4^ Department of Botany and Plant PathologyOregon State UniversityCordley Hall2701 SW Campus WayCorvallisOR97331USA; ^5^ Department of BiologyUniversity of WashingtonBox 351800SeattleWA98195‐1800USA; ^6^ Institut des Sciences de l’ÉvolutionUniversité Montpellier ‐ CNRS ‐ IRD ‐ EPHEPlace Eugène Bataillon ‐ CC 06534095Montpellier cédex 05France; ^7^ Department of Ecology and EvolutionUniversity of LausanneLe Biophore CH ‐ 1015LausanneSwitzerland; ^8^ Swiss Institute of BioinformaticsUniversity of LausanneQuartier Sorge ‐ Batiment Genopode 1015LausanneSwitzerland; ^9^ Duke Center for Genomic and Computational Biology (GCB)Duke University CIEMAS101 Science DriveDUMC Box 3382DurhamNC27708USA

**Keywords:** genetics, package, phylogenies, r, software

## Abstract

Genetic sequences of multiple genes are becoming increasingly common for a wide range of organisms including viruses, bacteria and eukaryotes. While such data may sometimes be treated as a single locus, in practice, a number of biological and statistical phenomena can lead to phylogenetic incongruence. In such cases, different loci should, at least as a preliminary step, be examined and analysed separately. The r software has become a popular platform for phylogenetics, with several packages implementing distance‐based, parsimony and likelihood‐based phylogenetic reconstruction, and an even greater number of packages implementing phylogenetic comparative methods. Unfortunately, basic data structures and tools for analysing multiple genes have so far been lacking, thereby limiting potential for investigating phylogenetic incongruence. In this study, we introduce the new r package *apex* to fill this gap. *apex* implements new object classes, which extend existing standards for storing DNA and amino acid sequences, and provides a number of convenient tools for handling, visualizing and analysing these data. In this study, we introduce the main features of the package and illustrate its functionalities through the analysis of a simple data set.

## Introduction

The constant improvement of sequencing technologies provides ever‐increasing amounts of genetic sequences for a wide range of organisms including viruses, bacteria and a variety of eukaryotes. As a consequence, organisms are more frequently sequenced for multiple genes, and full‐genome data are becoming increasingly common (Pettersson *et al*. 2009; Pareek *et al*. 2011). This is especially true for viruses and bacteria, whose smaller genomes allow for large collections of complete genome sequences to be assembled relatively easily (e.g. Harris *et al*. 2013; Weinert *et al*. 2015).

While it is tempting to treat multiple genes as a single evolutionary unit (de Queiroz & Gatesy 2007), a number of biological processes and statistical issues can lead to *phylogenetic incongruence*, causing different genes to exhibit distinct phylogenies (Wendel & Doyle 1998; Rokas *et al*. 2003; Pollard *et al*. 2006; Knowles 2009; Som 2015). Therefore, multiple gene data should ideally be first analysed separately to look for congruent genealogies and then possibly concatenated to derive a single phylogenetic tree. In practice, however, such an approach may not be trivial to implement.

Indeed, handling and analysing multiple gene alignments can be a daunting task. While the r software is growing as a platform of choice for phylogenetic analyses (Paradis *et al*. 2004; Jombart *et al*. 2010a; Kembel *et al*. 2010; Schliep 2011; Popescu *et al*. 2012), tools for handling and analysing multiple gene data are missing. In this study, we introduce *apex*, a new r package which fills this gap. *apex* implements new object classes for storing and handling multiple genes data. This package is fully integrated and compatible with existing r standards for phylogenetic reconstruction and makes the analysis of multiple genes as straightforward as for single genes. In the following, we provide an overview of these functionalities and illustrate the main features using a worked example.

## Functionalities

### New object classes

The main feature of *apex* is to provide classes for storing and handling multiple gene data. This is implemented through two novel formal (S4) classes, multidna and multiphyDat, which are respectively extensions of the DNAbin objects from the package *ape* (Paradis *et al*. 2004; Popescu *et al*. 2012), optimized for distance‐based methods, and phyDat objects from the package *phangorn* (Schliep 2011), better suited for parsimony and likelihood‐based approaches.

As in any formal class, the structure of multidna and multiphyDat is defined by a collection of predefined ‘slots’, each containing specific information such as the number of individuals in the data set, the various gene sequences and additional meta‐information. For the sake of simplicity, both classes have identical slots (Tables 1 and 2) and only differ in the way genetic sequences are stored internally: multidna uses bytes to code nucleotides (DNAbin objects, Table 1), while multiphyDat enumerates variable patterns in the sequences and can be used for DNA as well as amino acid sequences (phyDat objects, Table 2). In both cases, aligned sequences are stored inside a list (Tables 1 and 2), in which each element corresponds to a specific gene/locus. Besides storing genetic sequences, multidna and multiphyDat can also store labels for the individuals sequenced, as well as any metadata regarding the individuals or the genes of the data set (Tables 1 and 2).

**Table 1:** Content of multidna objects. The content of each slot can be accessed using ‘@[*slot name*]’, where ‘[*slot name*]’ is any of the values listed in the first column

Slot name	Data stored	Description
dna a	list of DNAbin matrices	A list of DNAbin matrices, each storing sequences of a given locus/gene; names are optional, and if provided, identify the genes; all matrices have the same individuals in rows, and nucleotide positions in columns
labels	character vector	A vector of labels for the individuals
n.ind	integer	The number of individuals in the data set
n.seq	integer	The total number of sequences, pooling all genes, and including gap‐only sequences
n.seq.miss	integer	The total number of gap‐only sequences
ind.info a	data.frame	A data.frame containing information on the individuals, where individuals are in rows
gene.info a	data.frame	A data.frame containing information on the genes, where genes are in rows

a Slots whose content is NULL when empty.

Both classes ensure that data are stored in a consistent way. When creating new multidna or multiphyDat objects, all individuals with at least one sequence are first enumerated and sorted alphanumerically. For each locus, gap‐only sequences are created for every missing individual, and new alignments containing all individuals, sorted identically, are created. As a result, the different gene alignments and their analyses can be readily compared, which greatly facilitates the assessment of congruence amongst the loci.

### Handling data

The fact that different gene data are stored in a consistent way also makes data manipulation easy. In both classes, we implemented matrix‐like subsetting using the syntax ‘[‘ operator. Assuming ‘x’ is a multidna or multiphyDat object, then ‘x[i, j]’ is a subset of ‘x’ where ‘i’ indicates the individuals to be kept, and ‘j’ the retained genes. This subsetting follows r standards and allows for vector of integers, characters or logical to be used, so that handling of multidna or multiphyDat objects should be as natural as usual objects (vectors and matrices).

In addition to the easy subsetting and reordering of individuals and genes, the generic function ‘concatenate’ can be used to merge several genes into a single alignment. By default, all genes in the object are used, but an optional argument permits to select which genes to include in the alignment.

### Importing and exporting data

Building on resources provided in *ape* (Paradis *et al*. 2004; Popescu *et al*. 2012), *phangorn* (Schliep 2011) and *adegenet* (Jombart 2008; Jombart & Ahmed 2011), *apex* provides functions to import and export data from and to a variety of formats (Table 2).

**Table 2:** Content of multiphyDat objects. The content of each slot can be accessed using ‘@[*slot name*]’, where ‘[*slot name*]’ is any of the values listed in the first column

Slot name	Data stored	Description
seq a	list of phyDat objects	A list of phyDat objects, each storing sequences of a given locus/gene; names are optional, and if provided identify the genes; all matrices have the same individuals in rows, and nucleotide positions in columns
type	character vector	A character string indicating the type of sequences (e.g. DNA, protein)
labels	character vector	A vector of labels for the individuals
n.ind	integer	The number of individuals in the data set
n.seq	integer	The total number of sequences, pooling all genes, and including gap‐only sequences
n.seq.miss	integer	The total number of gap‐only sequences
ind.info a	data.frame	A data.frame containing information on the individuals, where individuals are in rows
gene.info a	data.frame	A data.frame containing information on the genes, where genes are in rows

a Slots whose content is NULL when empty.

multidna and multiphyDat objects can both be created in r using the constructor ‘new(a, …)’, where ‘a’ is a character string indicating the class of the object (‘multidna’ or ‘multiphyDat’), and ‘…’ is a list of arguments passed to the constructor, the main one being a list of objects (character, DNAbin or phyDat matrices) storing DNA or amino acid sequences. However, it is likely that in most cases, genetic sequences of different loci will be stored in separate text files, using one file per gene. Three functions permit to import data from a list of files directly into *apex*. The functions ‘read.multidna’ and ‘read.multiFASTA’ build upon *ape*'s procedures to read data in interleaved, sequential, Clustal or FASTA format. In addition, the function ‘read.multiphyDat’ enables imports of amino acid sequences with interleaved, sequential or FASTA format. Note that in this case, the resulting multiphyDat object can no longer be converted to multidna, which is restricted to nucleotide sequences only.

Once imported in *apex*, data can also be converted to various formats (Table 3). Conversion from multidna to multiphyDat is implemented by multidna2multiphyDat, while the reverse operation is performed by multiphyDat2multidna. Single‐nucleotide polymorphisms (SNPs) can be extracted from the alignments and translated into a genind object, thereby providing access to a wide range of multivariate analyses (Jombart *et al*. 2008, 2009, 2010b). As an alternative, each unique gene sequence can be treated as a separate allele, generalizing the multilocus sequence type (MLST) approach which proved highly useful for classifying clonal organisms in microbiology (Maiden *et al*. 1998; Aanensen & Spratt 2005; Maiden 2006), and could be equally useful for large gene collections of nonclonal organisms.

### Accessors

A set of functions have been provided to access the slots in a multidna and multiphyDat objects. For example, the number of loci and their names can be obtained with the getNumLoci and getLocusNames functions, respectively. The setLocusNames function can also be used to set the names in the same way that the names, colnames and rownames functions work on standard r objects.

**Table 3:** Functions for importing and exporting data in *apex*

Function	Input	Output	Notes
read.multidna	Interleaved, sequential, clustal, fasta files	multidna	Based on read.dna (*ape* package)
read.multiFASTA	Fasta files	multidna	Based on read.FASTA (*ape* package)
read.multiphyDat	Interleaved, fasta	multiphyDat	Based on read.phyDat (*phangorn* package) can read amino acid sequences
multidna2multiphyDat	multidna	multiphyDat	
multiphyDat2multidna	multiphyDat	multidna	Only works for DNA sequences
multidna2genind	multidna	genind a	Extract either SNPs or haplotypes
multiphyDat2genind	multiphyDat	genind a	Extract either SNPs or haplotypes

a Base class for genetic markers in the *adegenet* package.

The number of sequences at each locus stored in the @dna slot can be obtained with the getNumSequences function. Counts of number of sequences for specific loci can be produced by providing a vector of their names to the loci argument of this function. By default, only sequences not composed entirely of gaps are counted; however, if the exclude.gap.only argument is FALSE, the number of all sequences is returned. Names of sequences at each locus can be obtained with the getSequenceNames function, which also has the exclude.gap.only argument that functions similarly.

The sequences themselves can be returned with the getSequences function. By default, this returns the list of DNAbin‐formatted sequences stored in the @dna slot. The sequences can be filtered for specific individuals and loci by providing a character vector to the ids and loci arguments, respectively. If only a single locus is returned and the simplify argument is TRUE (the default), then the return value is a single DNAbin object. If simplify is FALSE, the function will always return a list of DNAbin objects.

### Analysing data

As data visualization is often the first step in data analysis, we implemented a plot method for multidna objects, which permits to visualize the separate alignments simultaneously. This can be useful for a quick assessment of the quality of different alignments, or patterns of missing data (N or other ambiguous nucleotides) or alignment gaps. As each gene is an item of a list (the slots @dna and @seq, Tables 1 and 2), any operation usually carried out on a single gene can be applied to all genes using the base r function lapply. However, to facilitate further the analysis of sequences from multiple genes, we provide functions for applying the most common phylogenetic analyses to multiple gene data. The 17 different genetic distances implemented in *ape* can be computed using multidna objects, either separately for each gene or after concatenating them, using dist.multidna. The function getTree provides a wrapper for a number of phylogenetic reconstruction methods implemented in *ape*, including different versions of neighbour‐joining (Saitou & Nei 1987; Gascuel 1997) and minimum evolution trees (Desper & Gascuel 2002).

## Worked example

We illustrate a typical workflow of phylogenetic analysis in *apex* using data on eight species of New World chickadees (Aves: Paridae) typed on four nuclear loci (Harris *et al*. 2014). This data set is distributed with *apex* in two forms: a multidna object (data set ‘chickadees’) and as raw DNA alignments. We use the latter in this example. We assume all DNA alignments are stored as FASTA files in the working directory. After loading *apex*, we read and process the DNA alignments by selecting all files with ‘.fasta’ extension (this uses the base r command dir(pattern=".fasta")) using the *apex* function read.multiFASTA: 
> library(apex)
> x < - read.multiFASTA(dir(pattern=".fasta"))
> x
=== multidna ===
[32 DNA sequences in 4 genes]
@n.ind: 8 individuals
@n.seq: 32 sequences in total
@n.seq.miss: 8 gap-only (missing) sequences
@labels: 2340_50156.ab1 2340_50149.ab1
2340_50674.ab1 ...
@dna: (list of DNAbin matrices)
...



**Figure 1:**
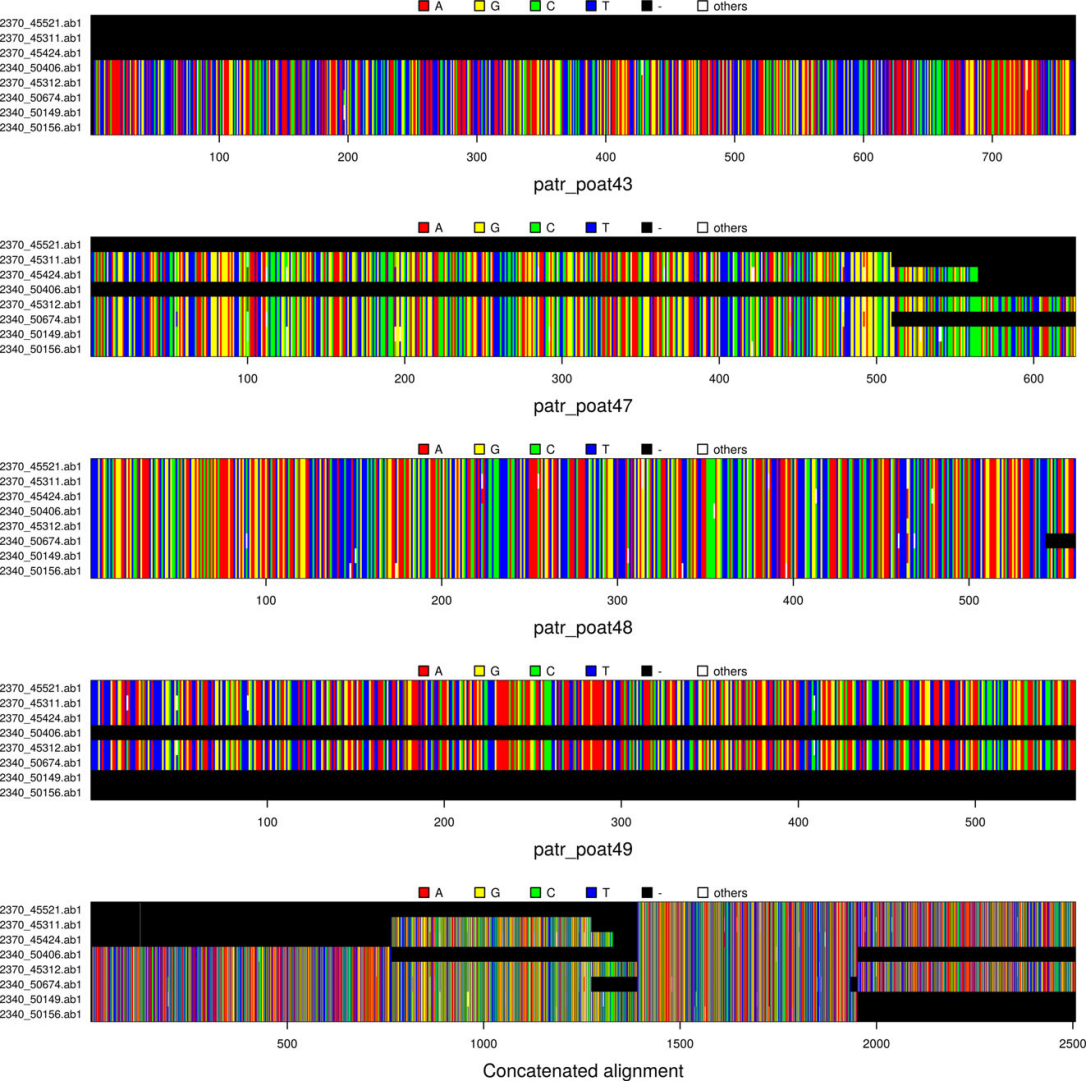
Individual and concatenated sequence alignments of chickadees data. The first four graphs are a plot of a multidna object containing DNA alignments for four different loci (patr_poat 43, 47, 48 and 49). The fifth graph displays the concatenated alignment.

Inspecting ‘x’, for example, by printing it (the full output, too long to be displayed, has been cut here) will indicate that the data consist of 32 DNA sequences coming from four different alignments which pooled together document 8 taxa. Eight gap‐only sequences were added by default for missing sequences, so that all DNAbin matrices in ‘x’ correspond to the same taxa (Fig. 1). The actual number of taxa present in each alignment is as follows: 
> getNumSequences(x)
patr_poat43 patr_poat47 patr_poat48 patr_poat49 
 5 6 8 5 



To ensure matching of taxa across genes, gap‐only sequences have been added where sequences were missing in ‘x’. For phylogenetic reconstruction, however, these gap sequences are best removed: 
> x.nogaps <- read.multiFASTA(dir
(pattern=".fasta"), gaps=FALSE)



Alignments can be visualized using the simple plot function (Fig. 1). A concatenated alignment (Fig. 1) can be obtained using the following: 
> y < - concatenate(x)



Individual phylogenies can be obtained for each locus using the following: 
> trees < - getTree(x.nogaps)



By default, getTree produces neighbour‐Joining trees using Hamming distances, using pairwise deletions, enforcing non‐negative branch lengths and ladderizing the trees. All of these can be customized using any distance and tree algorithms implemented in *ape* (Paradis *et al*. 2004), through arguments passed to getTree. Alternatively, a single tree can be obtained after concatenation of the alignments using the following: 
> tree <- getTree(x, pool=TRUE)



**Figure 2:**
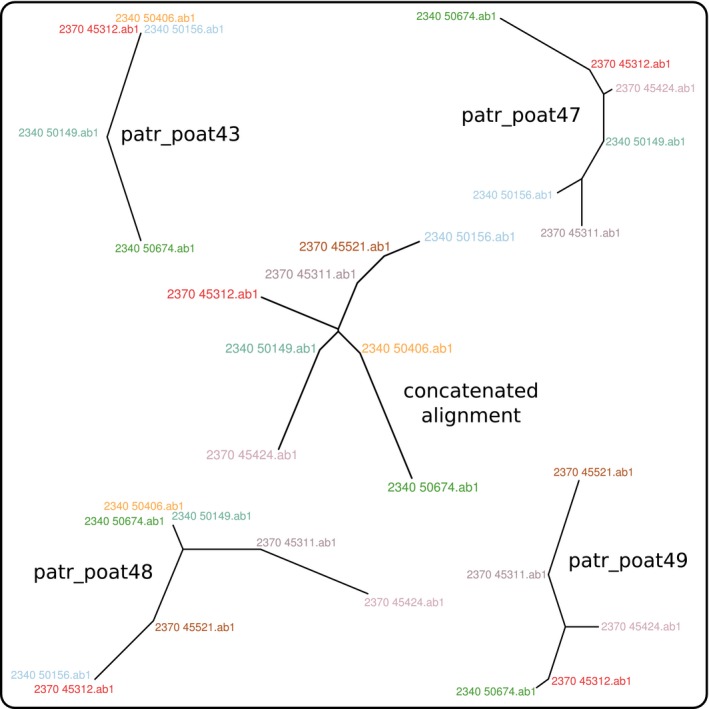
Phylogenies of the chickadees data. The function getTree was used to obtain individual phylogenies for each locus (patr_poat43, 47, 48 and 49), and from the concatenated alignment (central phylogeny). Each phylogeny is an unrooted, neighbour‐joining tree based on Hamming distances between DNA sequences. Taxa are identified using colours to facilitate comparison of the trees.

Despite the modest size of this data set, results show substantial phylogenetic incongruence amongst different loci (Fig. 2), stressing the need for considering several informative loci for achieving a more robust phylogenetic reconstruction (Harris *et al*. 2014).

## Discussion

Phylogenetic analysis of multiple gene data can be a daunting task, requiring substantial prior data processing (e.g. comparing sequenced taxa across different loci, handling missing data) and replication and comparison of the analyses over all loci. So far, this could only be achieved in r using multiple *ad hoc* scripts (e.g. Bryson *et al*. 2013; Harris *et al*. 2013; Grummer *et al*. 2015), thereby limiting the reproducibility and reliability of the analyses, and discouraging the investigation of potential phylogenetic incongruence (Som 2015). The aim of *apex* is to streamline this process and make phylogenetic reconstruction with multiple genes as straightforward as with a single locus.

To achieve this goal, *apex* implements new object classes which extend existing standards for DNA and amino acid sequences. It provides simple functions for importing multiple alignments data from classical formats and software, seamlessly taking care of re‐ordering and matching taxa across loci, identifying missing sequences, and optionally adding gap‐only sequences to ensure matching of taxa for all loci. The resulting objects can be handled easily using various accessor functions, procedures for subsetting data by taxa, locus or site, and for concatenating selected loci. Additionally, *apex* implements a series of wrappers for the visualization and analysis of multiple gene data and implements a generalization of sequence‐type polymorphism (Maiden *et al*. 1998; Aanensen & Spratt 2005; Maiden 2006) directly compatible with standard population genetics methods (Goudet 2005; Jombart 2008; Paradis 2010; Jombart & Ahmed 2011). This latter feature should be particularly useful for deriving fast classifications of organisms (e.g. ‘sequence types’ in bacteria) from large genomic data sets.

Arguably, dedicated statistical approaches are needed for an in‐depth investigation of phylogenetic discrepancies exhibited by multilocus data (Hillis *et al*. 2005; Knowles 2009; Jombart *et al*. 2015; Som 2015). By providing support for the representation, visualization and basic analysis of these data, *apex* should facilitate and hopefully encourage the development of new methods for exploring phylogenetic incongruence. As such, we believe it is a worthwhile addition to the growing platform for genetic sequence analysis in r.

T.J., F.A., K.S. and Z.K. developed the package. R.H. provided data sets. J.G., E.P. and H.L. provided advice for the package design. T.J., F.A. and H.L. wrote the manuscript.

## Software availability

The stable version of apex is released on the Comprehensive R Archive Network (CRAN):

http://cran.r-project.org/web/packages/apex/index.html

and can be installed in r by typing the following:

install.packages(apex)

The development version of *apex* is hosted on github:

https://github.com/thibautjombart/apex

*apex* is distributed under GNU Public Licence (GPL) version 2 or greater. It is fully documented in a vignette accessible by typing the following:

vignette(apex)
